# Co-infection with *Pseudomonas aeruginosa* and *Mycobacterium avium* complex in patients with bronchiectasis: coincidence or inevitability?

**DOI:** 10.3389/fmed.2026.1785156

**Published:** 2026-03-11

**Authors:** Xiaoni Zhou, Xiaohui Luo, Yuying Wu, Han Wang, Zhiyun Pan, Jun Wang, Xinhua Xiao, Minggui Lin, Zhi Yao

**Affiliations:** 1 Department of Pulmonary and Critical Care Medicine, Wuhan Pulmonary Hospital, Wuhan, Hubei, China; 2 Department of Infectious Diseases, Beijing Tsinghua Chang Gung Hospital, Tsinghua University, Beijing, China

**Keywords:** bronchiectasis, inflammation, microorganism, *Mycobacterium avium* complex, *Pseudomonas aeruginosa*

## Abstract

The burden of bronchiectasis is rapidly escalating worldwide, with its airway microbiome shifting from a “single-pathogen” paradigm to “multiple-pathogen coexistence.” Both *Mycobacterium avium* complex and *Pseudomonas aeruginosa* infections have been demonstrated to exacerbate airway destruction, yet they are rarely examined concurrently. Emerging evidence suggests these pathogens exhibit a long-term coexistence pattern within the same patient, with one dominating when the other recedes. Furthermore, existing studies indicate that the disease burden in coinfected patients is higher than in those with either pathogen alone. However, the specific competitive and synergistic interactions between *M. avium* complex and *P. aeruginosa* in bronchiectasis patients remain poorly recognized, posing substantial therapeutic challenges. This review summarizes current understanding of the epidemiology and clinical manifestations of *M. avium* complex and *P. aeruginosa* co-infection in patients with bronchiectasis, along with potential mechanisms of microbial interaction between the two pathogens.

## 1 Introduction

In recent years, the increasing prevalence of bronchiectasis has become a significant public health issue due to its substantial disease burden and impact on patients’ quality of life. As an important cause of chronic respiratory failure and recurrent acute exacerbations worldwide, its heterogeneous clinical manifestations are closely associated with the persistent presence of pathogenic microorganisms in the airway (1). With advancements in microbial detection technologies, we have increasingly recognized that patients’ airways are often not infected or colonized by a single pathogen, but rather by multiple pathogens that coexist over the long term (2, 3). Nontuberculous mycobacteria (NTM) constitute a large bacterial group excluding *Mycobacterium tuberculosis* and *Mycobacterium leprae*, with over 190 distinct bacterial species identified to date (4). Based on growth rate, NTM are classified into fast-growing mycobacteria, represented by the *Mycobacterium abscessus* complex, and slow-growing mycobacteria, primarily the *Mycobacterium avium* complex (5). According to existing reports, *M. avium* complex is the most common NTM species causing pulmonary infections in different countries and regions (6), with *M. avium* complex infections in South Korea reaching as high as 88% (7).

For a long time, a potential association has been identified between *M. avium* complex infection and bronchiectasis. Still, it has also sparked some controversy: does airway structural damage lead to *M. avium* complex infection, or does *M. avium* complex infection itself cause bronchiectasis? Over the past two decades, *Pseudomonas aeruginosa*, due to its biofilm-forming capacity and multidrug resistance, has been identified as the primary culprit for accelerating the decline in lung function and increased frequency of acute exacerbations in patients with bronchiectasis (8–11). Although the epidemiology, pathological mechanisms, and clinical outcomes of bronchiectasis and infections by both pathogens have been relatively widely discussed, co-infection has long been neglected. Increasing research has revealed that in patients with bronchiectasis, the culture results of *P. aeruginosa* and *M. avium* complex exhibit a dynamic interplay, with one growing as the other decreases, and a long-term coexistence pattern (12). However, is this dynamic phenomenon merely a coincidental increase in laboratory detection rates, or does it reflect an inherent biological connection between the two pathogens? Additionally, the clinical burden on patients with co-infection is far greater than previously imagined. Some retrospective studies have indicated that patients with bronchiectasis co-infected with *P. aeruginosa* and NTM (particularly *M. avium* complex) have a higher frequency of acute exacerbations and a shorter interval between the first hospitalization compared to those with NTM infection alone (13–15). However, whether *P. aeruginosa* is the primary driving force or both pathogens contribute synergistically remains to be further investigated. Despite recent advances in the pathophysiological mechanism of bronchiectasis co-infection with *P. aeruginosa* and *M. avium* complex, challenges remain due to limited understanding as well as the complexity of co-infection itself.

In this review, we summarize the clinical status of co-infection with *M. avium* complex and *P. aeruginosa* in bronchiectasis, as well as the interaction mechanisms between the pathogens and the host, with a view to providing possible directions for future research.

## 2 Epidemiology

*Pseudomonas aeruginosa* has long been recognized as the most important pathogen associated with bronchiectasis. Meanwhile, the prevalence of NTM in patients with bronchiectasis ranges from 2.0% to 37.0% (16–18), although there is significant variation, the overall trend is upward globally (19–21). Many studies have shown that NTM lung disease often coexists with bronchiectasis, and *M. avium* complex infection may also become a significant cause of bronchiectasis (22, 23). Aksamit et al. (24) evaluated 1,826 patients in the US Bronchiectasis Study Registry from 2008 to 2014 and found that 63% of patients had NTM infection, dominated by *M. avium* complex. A national epidemiological survey from Japan showed that approximately 74% of non-cystic fibrosis bronchiectasis patients had concurrent NTM lung disease, with *M. avium* complex being the most prevalent (25). Recently, a meta-analysis covering 41 countries worldwide and including 98 studies also pointed out that *P. aeruginosa* was the most frequently isolated bacterium in adult patients with non-cystic fibrosis bronchiectasis at different stages of the disease, while *M. avium* complex was the most frequent isolated mycobacterium (26).

Thus, the *P. aeruginosa* and *M. avium* complex exhibit a dual high prevalence pattern in bronchiectasis, and their co-infection in bronchiectasis has also garnered significant attention in recent years. Two large-scale retrospective studies from different regions showed that the proportion of baseline co-infection with NTM and *P. aeruginosa* in patients with bronchiectasis ranged from 13.7% to 19% (27, 28). A single-center cross-sectional study found that 7.8% of patients with *M. avium complex* lung disease had concurrent chronic *P. aeruginosa* infection (14). Fujita et al. retrospectively analyzed 275 patients with *M. avium* complex lung disese and revealed that 45 cases had *P. aeruginosa* co-infection, with 35 cases classified as chronic co-infection (interval ≥ 3 months and detection of *P. aeruginosa* in 2 or more sputum cultures). Interestingly, the study showed that among the 35 patients with chronic co-infection, only 25.7% (*n* = 9/35) had positive sputum cultures for both *M. avium* complex and *P. aeruginosa*, 75% (*n* = 18/24) developed *P. aeruginosa* sputum culture positivity during *M. avium* complex treatment, and 93.1% (*n* = 27/29) of patients isolated *P. aeruginosa* after *M. avium* complex sputum culture turned negative (12). Coincidentally, Urabe et al. (29) also observed that 58.3% of patients with *M. avium* complex lung disease tested positive for *P. aeruginosa* after initiating treatment. The study noted that in the co-infection group, *M. avium complex* treatment not only inhibited the growth of *M. avium* complex and the colonization of macrolide-sensitive pathogens, but also created opportunities for the proliferation of macrolide-resistant bacteria such as *P. aeruginosa*. Collectively, these data indicate that patients are more susceptible to infection and chronic colonization with *P. aeruginosa* after initiating treatment and achieving conversion of *M. avium* complex. Meanwhile, Kamata et al. (14) reported that patients with chronic *P. aeruginosa* infection and *M. avium* complex lung disease exhibited lower rates of NTM-positive sputum cultures. In addition, several studies have shown that patients with NTM infection, including those with cystic fibrosis and bronchiectasis, have lower rates of chronic *P. aeruginosa* infection than those without NTM infection (24, 30).

In summary, these studies suggest that *P. aeruginosa* and *M. avium* complex may imply some special association, with the two pathogens potentially exhibiting dynamic competitive inhibition and ecological displacement. In the real world, the incidence of co-infection may be higher than the existing data.

## 3 Clinical manifestations and prognosis

**Figure 1:**
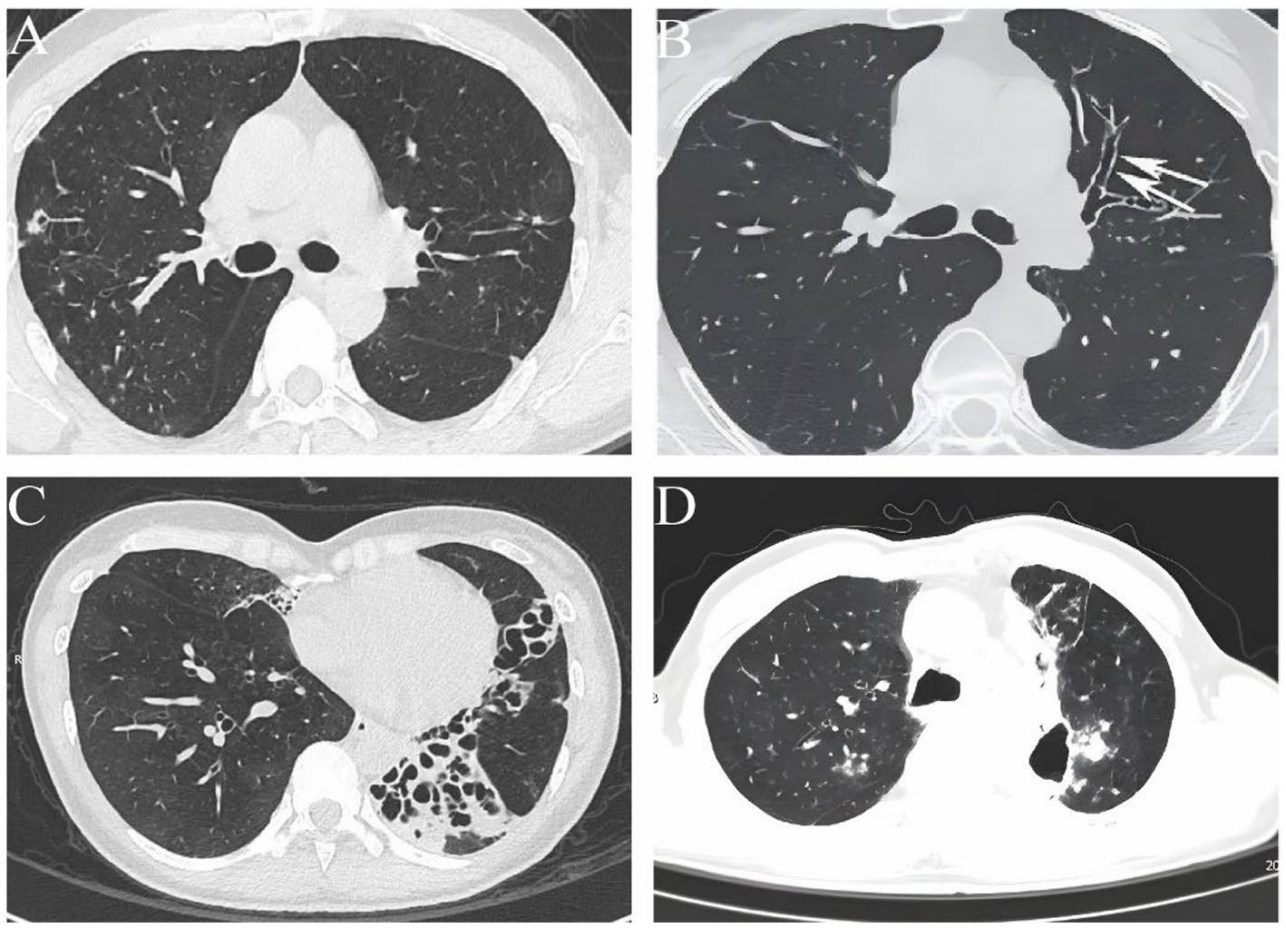
Abnormal pulmonary computed tomography images in patients with bronchiectasis complicated by *M. avium* complex infection. (**A)** Nodular lesions and bronchiectasis. **(B)** Cylindrical bronchiectasis. (**C)** Cystic bronchiectasis. (**D)** Thin-walled cavity.

Bronchiectasis commonly presents clinically with chronic or persistent cough, production of large amounts of purulent sputum, intermittent hemoptysis, with or without varying degrees of symptoms such as dyspnea and respiratory failure (31). When combined with *P. aeruginosa* infection, sputum characteristics are often changed, with most patients characterized by yellowish-green purulent sputum, increased sputum volume, and greater viscosity (32). Some patients may develop new episodes of hemoptysis (33). Chest CT scans reveal bronchial wall thickening, mucus plug filling, tree-in-bud signs, and patchy or nodular areas of increased density in the lung parenchyma (34). Studies have shown that these patients have wider lung lesions and more involved lung lobes (35), while long-term chronic infection can accelerate the progression of bronchiectasis (36), and is closely associated with declining lung function (37), increased frequency of acute exacerbations, and higher hospitalization rates (38). Furthermore, *P. aeruginosa* represents an independent risk factor for mortality in bronchiectasis (39). Unlike *P. aeruginosa* infection, patients with bronchiectasis complicated by *M. avium* complex infection share most clinical features with uninfected patients, and some patients often present with symptoms similar to *Mycobacterium tuberculosis* infection, such as coughing, sputum production, hemoptysis, and systemic wasting symptoms like fatigue, night sweats, and weight loss (40, 41). However, these clinical features lack specificity, and due to the insidious onset, they are often misdiagnosed as pulmonary tuberculosis or exacerbation of pre-existing bronchiectasis. Currently, high-resolution CT imaging is the primary distinguishing feature. The majority of patients exhibit chest radiographic findings showing involvement of the middle lobe of the right lung and the lingular segment of the left upper lobe, presenting as columnar or cystic bronchiectasis, accompanied by multiple micronodules, infiltrative shadows, and tree-in-bud signs, with some cases showing thin-walled cavities (41–44). The relevant imaging characteristics are clearly shown in Figure 1. Compared to *P. aeruginosa*, whether NTM infections affect the prognosis of patients with bronchiectasis remains a matter of debate. Frajman et al. (45) found that bronchiectasis patients with positive NTM cultures (46.8% of which were *M. avium* complex) experienced more frequent acute exacerbations in the year preceding bronchoscopy and demonstrated poorer lung function. These findings are largely consistent with the conclusions of a retrospective study from China (41). However, another study involving 2,634 patients with bronchiectasis followed for ≥5 years indicated that patients with and without NTM infection had similar rates of acute exacerbations, hospitalizations, and mortality over 5 years. Although this may be partly related to higher antibiotic usage among NTM patients (27), similar findings were also reported by Japanese researchers (46).

**Figure 2:**
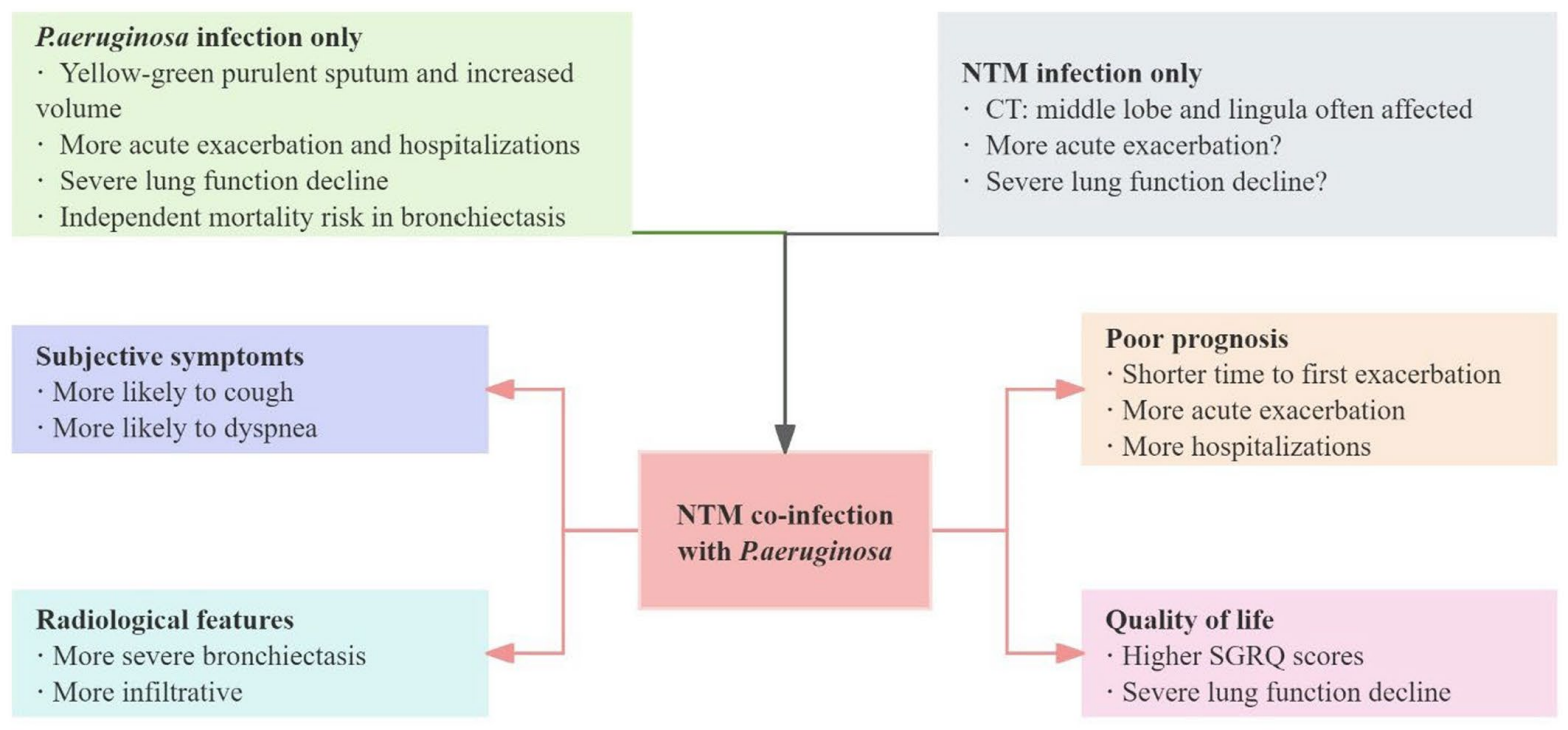
Clinical impact of non-tuberculous mycobacteria and *P. aeruginosa* infections, both as single infections and co-infections (mainly refers to *P. aeruginosa* co-infection following non-tuberculous mycobacteria infection).

Bronchiectasis coexisting with *P. aeruginosa* and *M. avium* complex infection does not present with specific clinical symptoms, with cough and sputum production remaining the most common manifestations. Moon et al. (15) noted that NTM lung disease patients with *P. aeruginosa* infection experienced more frequent coughing and sputum production (70% *vs.* 38%; *p* = 0.008) and a higher incidence of dyspnea (30% *vs*. 13%; *p* = 0.047). In this study, nearly all patients with NTM infection developed bronchiectasis (*n* = 169/180, 94%), and 84% (*n* = 151/180) of patients had *M. avium* complex infection. Of particular concern, bronchiectasis patients with *M. avium* complex infection who also develop *P. aeruginosa* face a heavier clinical burden and worse prognosis. Patients with *M. avium* complex infection complicated by chronic *P. aeruginosa* infection had significantly higher St. George’s Respiratory Questionnaire scores than those with *M. avium complex* infection alone, suggesting that co-infected patients experience a striking decline in quality of life, furthermore, these patients also exhibited more severe bronchiectasis (14). Wang et al. pointed out that *M. avium* complex combined with bacterial infections (mainly *P. aeruginosa* and *Staphylococcus aureus*) is more common in patients with cystic fibrosis and bronchiectasis, and chest CT images tend to more infiltrative shadows in patients with co-infections. Unfortunately, due to insufficient sample size, this study did not further analyze the imaging manifestations of *M. avium* complex co-infection with single different bacterial infections (47). In addition, in the initial stage of patients diagnosed with *M. avium* complex lung disease, the time to first acute exacerbation of bronchiectasis is shorter when concurrent *P. aeruginosa* infection occurs (48). However, whether NTM (including *M. avium* complex) produces similar effects in patients with bronchiectasis who have *P. aeruginosa* infection at baseline remains debatable. A single-center retrospective study involving 96 patients with non-cystic bronchiectasis revealed that patients with co-infection of NTM (primarily the *M. avium* complex) and *P. aeruginosa* had significantly lower forced expiratory volume in 1 s and forced vital capacity compared to patients with single infection of either NTM or *P. aeruginosa*, as well as had a notably higher frequency of acute exacerbations, emergency department visits, and hospitalizations (13). As the impact of NTM infection on bronchiectasis remains uncertain, definitive conclusions cannot be drawn. Based on the dynamic balance between NTM and *P. aeruginosa* in existing studies, could the negative effects of NTM on bronchiectasis be offset by the benefits of reduced *P. aeruginosa*? These data suggest that we should pay attention to the follow-up of patients with bronchiectasis complicated by *M. avium* complex infection, especially by frequently performing sputum routine bacterial culture tests, be alert to co-infection with *P. aeruginosa* if the patient’s clinical symptoms deteriorate, and initiate targeted anti-infective treatment as early as possible. Additionally, we present the clinical manifestations and prognosis of NTM and *P. aeruginosa* infections in Figure 2.

## 4 Mechanisms of microbial interactions

Increasing research indicates that the complex interactions among airway microbiota are a potential pathological mechanism driving the onset and progression of various chronic infections, such as chronic obstructive pulmonary disease and bronchiectasis (49–52). As a major contributing factor to bronchiectasis, enhanced microbial load not only triggers acute exacerbations of the disease but also produces toxins and metabolites that induce persistent inflammatory reactions, creating a vicious “microbe-inflammation” cycle. Nontuberculous mycobacteria have often been overlooked in the diagnosis and treatment of bronchiectasis. *P. aeruginosa*, as a key driver of infection in bronchiectasis, contributes to a substantial disease burden when co-infected with NTM. However, the interaction between *M. avium* complex (the most common Nontuberculous mycobacterium) and *P. aeruginosa* is very complex.

Research has shown that this co-infection activates the toll-like receptor (TLR) 2/ TLR4 signaling pathway synergistically, inducing dendritic cells (DCs) to highly express interleukin (IL)-10 and thereby suppressing T cell function (53), while simultaneously stimulating the explosive release of IL-1β (54), forming a unique “immune suppression-inflammatory damage” cycle that both weakens the host defensive capabilities and exacerbates tissue destruction, creating favorable conditions for the chronic colonization of pathogens. Notably, biofilms may play a key role in co-infection by regulating competitive and symbiotic relationships between microorganisms, thereby influencing the course of infection. Although direct evidence is currently limited, *in vitro* studies on *P. aeruginosa* and *Mycobacterium abscessus* co-infection suggest that microbial interactions in biofilm environments exhibit spatiotemporal specificity, and antibiotic intervention may paradoxically promote the transition of NTM into a persister state (55). These findings not only reveal that co-infection is an ecological strategy for microorganisms to actively adapt to the host environment but also suggest that the biofilm-mediated dynamic balance of “antagonism-compromise” may be an important factor determining the outcome of infection.

### 4.1 Co-infection with *M. avium* complex and *P. aeruginosa* induces “immune suppression-inflammatory storm”

In chronic pulmonary infections, co-infection with NTM and *P. aeruginosa* forms a “pathological community” that both inhibits clearance and continuously damages lung tissue through cross-interference of signaling pathways, imbalance of cytokine networks, and metabolite-mediated remodeling of the immune microenvironment. Kim et al. investigated the phenotypic and functional changes in DCs following *Mycobacterium avium* subspecies *hominissuis* (MAH) infection in response to various toll-like receptor agonists (simulating co-infection conditions), as well as subsequent T cell responses to reveal that lipopolysaccharide (LPS), via the TLR4 signaling pathway, synergizes with MAH induced TLR2 signaling, leading to significant IL-10 production in MAH infected DCs, whereas actual co-infection with MAH and *P. aeruginosa* results in an explosive increase in IL-10 expression. Additionally, IL-10, which is highly expressed in LPS/MAH co-infected DCs, upregulates programmed death-ligand 1 by decreasing major histocompatibility complex (MHC) class II molecule expression and MHC class II-antigen presentation, subsequently inhibits CD4^+^ T cell proliferation, reduces interferon-*γ* (IFN-γ) and IL-2 production, and ultimately stimulates the generation of tolerogenic DCs (53). It is worth noting that these tolerogenic DCs are not completely functionally deficient; rather, compared to inflammatory DCs, tolerogenic DCs express fewer co-stimulatory molecules on their surface, secrete fewer pro-inflammatory factors, but produce higher levels of the anti-inflammatory cytokine IL-10 (56, 57). This seems to indicate that co-infection promotes the formation of a local immunosuppressive microenvironment. Although MAH may activate the initial immune response through TLR2 activation, under co-infection conditions, the synergistic activation of TLR4 instead triggers an immunosuppressive program, thereby weakening the body’s ability to clear the *P. aeruginosa* and *M. avium* complex. This may also provide an immune evasion mechanism for the chronic colonization and persistent infection of both pathogens.

**Figure 3:**
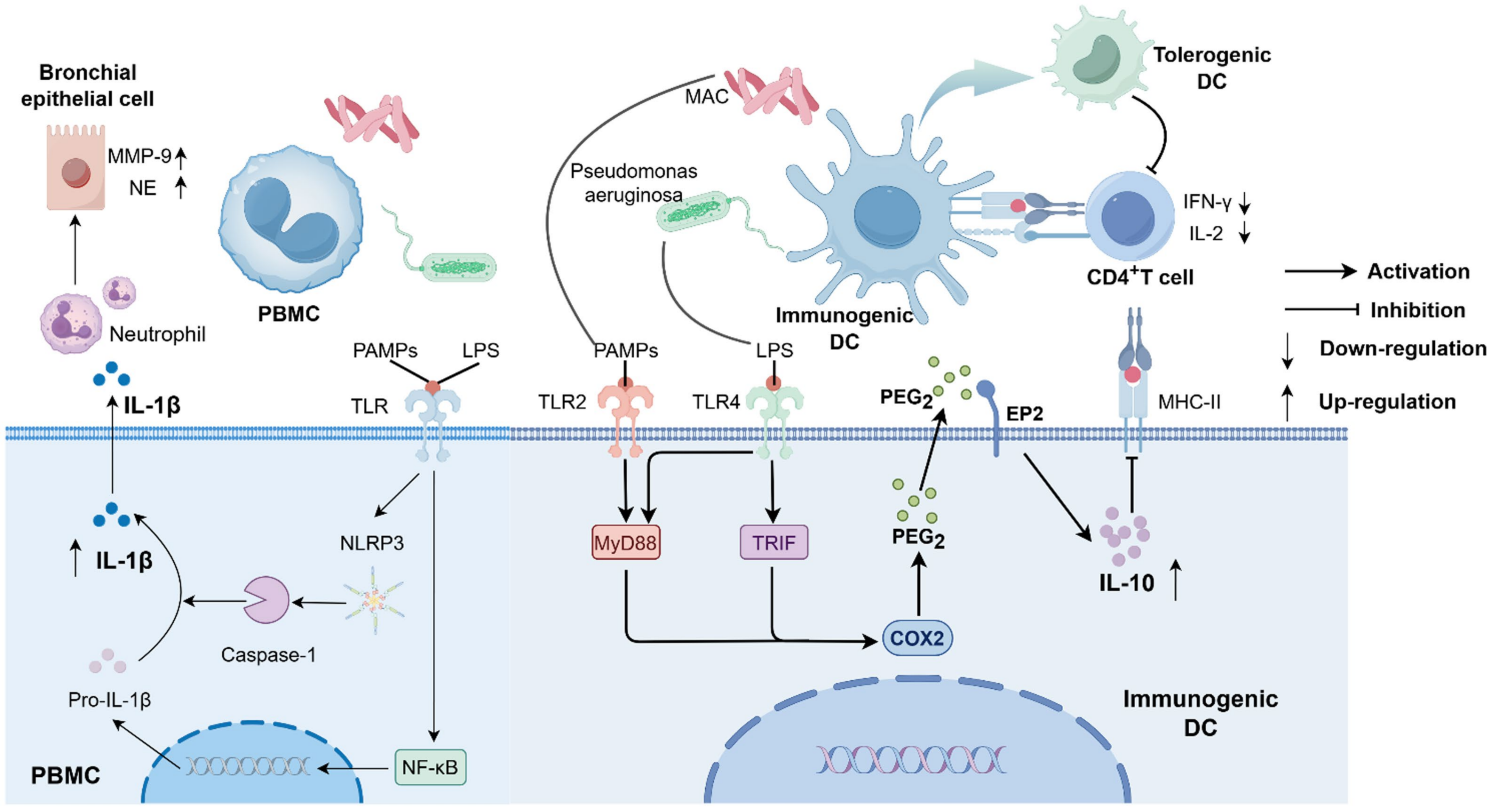
The *P. aeruginosa* and *M. avium* complex co-infect dendritic cells and human peripheral blood mononuclear cells, inducing the massive expression of IL-10 and IL-1β.

However, the flip side of co-infection is an uncontrolled inflammatory storm. Carazo-Fernández et al. (54) co-cultured NTM strains (including *Mycobacterium avium*, *Mycobacterium intracellulare*, *Mycobacterium kansasii*, and *Mycobacterium gordonae*) and *Mycobacterium tuberculosis* isolated from sputum with human peripheral blood mononuclear cells. After 18 h, *P. aeruginosa* was added, and the co-culture was continued for another 18 h. They found that the *M. avium* complex co-infected with *P. aeruginosa* significantly stimulated the production of IL-1β in human peripheral blood mononuclear cells. IL-1β is a significant pro-inflammatory cytokine produced by cells of the innate immune system, induced by pathogen-associated molecular patterns, and released after processing by caspase-1 via the NLRP3 inflammasome. While it can recruit neutrophils and amplify the inflammatory cascade, temporarily suppressing the spread of invading pathogens (58, 59), its overexpression may lead to uncontrolled inflammation, ultimately triggering the release of matrix metalloproteinase-9 and neutrophil elastase by bronchial epithelial cells, which degrade elastin and collagen fibers, leading to structural damage of the bronchial wall (60, 61). This is also a core mechanism in the progression of bronchiectasis (62). Notably, this synergistic effect is pathogen specific. Studies have found that *Mycobacterium kansasii* and *Mycobacterium gordonae* do not possess the ability to promote *P. aeruginosa* growth or stimulate the excessive production of IL-1β (54). In conclusion this seemingly contradictory phenomenon may imply a unique pathophysiological mechanism underlying co-infection between *M. avium* complex and *P. aeruginosa*: on the one hand, IL-10 mediated immune suppression impedes the effective clearance of pathogens; on the other hand, IL-1β driven excessive inflammation leads to tissue damage, promoting the formation of bronchiectasis, which further provides an ecological niche for *P. aeruginosa* infection or even colonization. Perhaps this “destruction-escape-circulation” is the core reason for the repeated isolation of *M. avium* complex and *P. aeruginosa* in the sputum of bronchiectasis patients. We describe a diagram based on the current potential mechanisms (Figure 3).

The interaction between microorganisms and their metabolites with the host’s innate immune system is an important factor in maintaining homeostasis. When we extend our perspective to the microbiome-metabolism-immunity axis, the chronicity of co-infections gains a more comprehensive explanation. Studies have shown that the diversity of lung microbiota in NTM patients is significantly reduced, exhibiting a unique microbial community dominated by *Pseudomonas* and *Staphylococcus aureus*, accompanied by changes in metabolite levels (63). Li et al. (64) utilized metagenomics and metabolomics to discover that *M. avium* complex, *Mycobacterium abscessus*, and *P. aeruginosa* jointly participate in the metabolic pathway converting indole to tryptophan via tryptophan synthase in NTM patients, leading to elevated levels of kynurenine, which can evade immune surveillance by inducing regulatory IDO1 + DCs, thereby promoting the expansion of regulatory T cells and inhibiting the response of T helper 17 cell (65), forming a unique immunosuppressive microenvironment for NTM infection and evading immune surveillance. This metabolite-mediated immune dysregulation allows the co-infection of NTM with *P. aeruginosa* to transcend simple virulence addition, evolving into an active ecological strategy to reshape the host microbiome for long-term coexistence.

### 4.2 Biofilms may be an important mechanism for “retention and competitive inhibition” in co-infections

Currently, research on the structure and function of *Mycobacterium avium* biofilms is still limited. Existing evidence suggests that glycopeptidolipids (GPL) and extracellular DNA (eDNA) play a significant role in the formation of *Mycobacterium avium* biofilms (66, 67). The formation of biofilm initially relies on GPL-mediated sliding movement (68), and is also co-regulated by divalent cations (such as Ca^2+^, Mg^2+^, Zn^2+^), carbon sources, and quorum sensing systems (69). eDNA, as a part of the biofilm matrix, undergoes structural disruption upon exposure to DNase, suggesting its irreplaceable role in maintaining biofilm structural integrity (70). Additionally, studies indicate that the production and release of eDNA are also dependent on quorum sensing mechanisms (71). In summary, biofilms confer *M. avium* complex resistance to chlorine, ozone, and ultraviolet light *in vitro* (72, 73) and significantly enhance its adhesion and invasion of bronchial epithelial cells *in vivo* (74). The main components of *P. aeruginosa* biofilms primarily include extracellular polysaccharides (Marine algal polysaccharides, Pel, and Psl) and eDNA (75). *P. aeruginosa* facilitates bacterial aggregation and induces the release of eDNA by up-regulating the synthesis of rhamnose polysaccharide and other compounds through extracellular polysaccharides Psl/Pel and quorum sensing (76). Extracellular polysaccharides and eDNA interact to form an eDAN-Psl/Pel fiber network, providing *P. aeruginosa* with a robust biofilm scaffold (77). Interestingly, WANG et al. noted that the Psl of *P. aeruginosa* can also interact with the DNA of *Staphylococcus aureus* (78), suggesting that *P. aeruginosa* can utilize the DNA of other organisms as a scaffold to form its own community. When co-existing with *M. avium* complex, it may also inhibit the growth of the latter through this mechanism. However, in an *in vitro* model simulating *in vivo* biofilm respiratory tract infections, ROSE’s research team found that the cell-free components of MAH biofilms strongly stimulate THP-1 human mononuclear phagocytes to produce inflammatory factors such as tumor necrosis factor alpha (TNF-*α*) and reactive oxygen species, causing the body to rapidly initiate a Th1-type immune response during the initial infection of MAH. However, the high expression of TNF-α also triggers early apoptosis of macrophages, providing an immune evasion strategy for persistent MAH infection (79). According to the immune bias mechanism (80), MAH infection drives the body to form a Th1-type immune polarization microenvironment dominated by TNF-α, which suppresses the *P. aeruginosa*-dependent Th2/Th-17 inflammatory niche (81). This seems to explain why, despite the ability of the *M. avium* complex and *P. aeruginosa* to form a unique chronic persistent co-infection symbiosis, patients with *M. avium* complex sputum culture positivity rarely exhibit concurrent *P. aeruginosa* positivity, and vice versa.

Unfortunately, there is currently no research on the mechanisms of biofilm interactions between the *M. avium* complex and *P. aeruginosa* in co-infections. At present, most studies on biofilm mechanisms associated with NTM co-infected with *P. aeruginosa* focus on *Mycobacterium abscessus*. Idosa et al. found that *P. aeruginosa* specifically inhibits the growth of *Mycobacterium abscessus* in biofilms, but this effect is not observed in planktonic co-cultures. Additionally, the growth of *Mycobacterium abscessus* is significantly inhibited only on days 1–2 of co-culture, with growth stabilizing by days 3–6 (82). This suggests that competitive inhibition and dynamic equilibrium in co-infection are closely related to biofilms, a finding consistent with the research by Rodriguez-Sevilla et al. (55). Furthermore, this antagonistic effect does not depend on traditional pathways known as contact-dependent/independent mechanisms (such as quorum sensing systems, secretion systems, motility, and iron uptake). It is noteworthy that this inhibitory effect lacks pathogen specificity, as the same phenomenon was observed in *Mycobacterium smegmatis* (82). Whether it can similarly inhibit other NTM, such as the *M. avium* complex, requires further research to verify. In an *in vitro* dual-species biofilm model of *P. aeruginosa* and *Mycobacterium abscessus*, researchers also showed that selective antibiotics targeting of *P. aeruginosa* promote the growth and colonization of *Mycobacterium abscessus*, ultimately exacerbating the resistance of the infection (55). Although Gupta et al.’s study found that *P. aeruginosa* can inhibit *Mycobacterium abscessus* without relying on biofilm, the study also revealed that *Mycobacterium abscessus* has its own survival strategies, including entering a non-replicative quiescent state through transcriptional reprogramming, downregulating metabolic and cell division genes, upregulating lipid storage and virulence factors, and simultaneously degrading *P. aeruginosa*’s quorum sensing signals and detoxification metabolites to adapt to competition (83, 84). In conclusion, these studies provide us with insights into the co-infection of *P. aeruginosa* and *M. avium* complex, and future research is expected to explore their potential pathological mechanisms.

## 5 Conclusion

Bronchiectasis patients co-infected with *M. avium* complex and *P. aeruginosa* have become a significant challenge in clinical diagnosis and treatment. Based on its non-specific clinical symptoms and poor prognosis, it is crucial to actively investigate the underlying mechanisms and implement precise diagnostic and therapeutic strategies. Currently, both *P. aeruginosa* and *M. avium* complex are highly prevalent in bronchiectasis. Whether due to improved detection rates, antibiotic use, or objective reality, an increasing number of studies indicate that the incidence of co-infection among these two pathogens is not low, and clinically, they often show a dynamic interplay of “one rising as the other falls,” suggesting potential competitive inhibition and ecological replacement. However, the underlying mechanisms remain unclear. This paper summarizes the existing research and proposes potential mechanisms, including the “immunosuppression-inflammatory damage” cycle, biofilm interactions, and ecological microenvironment construction. Unfortunately, relevant clinical studies are still scarce, and existing mechanism studies have certain limitations, as they are unable to simulate the real *in vivo* environment. In summary, future research should establish long-term, multi-center prospective cohorts under unified diagnostic criteria to dynamically quantify the actual incidence rate of co-infection between *M. avium* complex and *P. aeruginosa* in patients with bronchiectasis and its impact on disease progression, explore more precise microbial detection technologies, and deeply investigate the interaction mechanisms of co-infection between *M. avium* complex and *P. aeruginosa*. This will help identify potential therapeutic targets, optimize co-infection treatment strategies, improve patient outcomes, and reduce the social burden.
